# The ontogeny of naïve and regulatory CD4^+^ T-cell subsets during the first postnatal year: a cohort study

**DOI:** 10.1038/cti.2015.2

**Published:** 2015-03-27

**Authors:** Fiona M Collier, Mimi L K Tang, David Martino, Richard Saffery, John Carlin, Kim Jachno, Sarath Ranganathan, David Burgner, Katrina J Allen, Peter Vuillermin, Anne-Louise Ponsonby

**Affiliations:** 1 Child Health Research Unit and Barwon Biomedical Research, University Hospital, Barwon Health, Geelong, Victoria, Australia; 2 School of Medicine, Deakin University, Waurn Ponds, Victoria, Australia; 3 Murdoch Childrens Research Institute, Parkville, Victoria, Australia; 4 The Royal Childrens Hospital, Parkville, Victoria, Australia; 5 The University of Melbourne, Parkville, Victoria, Australia

## Abstract

As there is limited knowledge regarding the longitudinal development and early ontogeny of naïve and regulatory CD4^+^ T-cell subsets during the first postnatal year, we sought to evaluate the changes in proportion of naïve (thymic and central) and regulatory (resting and activated) CD4^+^ T-cell populations during the first postnatal year. Blood samples were collected and analyzed at birth, 6 and 12 months of age from a population-derived sample of 130 infants. The proportion of naïve and regulatory CD4^+^ T-cell populations was determined by flow cytometry, and the thymic and central naïve populations were sorted and their phenotype confirmed by relative expression of T cell-receptor excision circle DNA (TREC). At birth, the majority (94%) of CD4^+^ T cells were naïve (CD45RA^+^), and of these, ~80% had a thymic naïve phenotype (CD31^+^ and high TREC), with the remainder already central naïve cells (CD31^−^ and low TREC). During the first year of life, the naïve CD4^+^ T cells retained an overall thymic phenotype but decreased steadily. From birth to 6 months of age, the proportion of both resting naïve T regulatory cells (rTreg; CD4^+^CD45RA^+^FoxP3^+^) and activated Treg (aTreg, CD4^+^CD45RA^−^FoxP3^high^) increased markedly. The ratio of thymic to central naïve CD4^+^ T cells was lower in males throughout the first postnatal year indicating early sexual dimorphism in immune development. This longitudinal study defines proportions of CD4^+^ T-cell populations during the first year of postnatal life that provide a better understanding of normal immune development.

Following birth and during the first postnatal year, the infant's immune system must rapidly develop in order to negotiate the infectious milieu of the extrauterine environment as well as to establish tolerance to abundant nonpathogenic antigens. The development of immune phenotypes during this time may define the risk of infective illnesses or program the subsequent risk of immune-related disease.^1, 2^ Several studies have described the proportions of basic lymphocyte subsets at birth,^3, 4^ over the first year of life,^5, 6^ and during childhood and adolescence,^7^ but there are limited data regarding the longitudinal natural development of these subsets or potential gender differences during the first postnatal year. At birth, thymic (T) cells, derived from precursors that have undergone both positive and negative selection and have a low affinity for self-peptides, comprise approximately half of the circulating lymphocyte population.^8^ The CD4 surface marker denotes two subsets: T helper cells that have a central orchestrating role in the adaptive immune responses to various infectious agents,^9^ and a smaller subset of T regulatory cells (Treg) with a vital role in immune homeostasis.^10^

In cord blood, the phenotype of the neonatal CD4^+^ compartment is primarily naïve (positive for CD45RA antigen), and thought to comprise ‘recent thymic emigrants' or ‘thymic naïve' T cells.^11, 12^ These thymic naïve T cells in cord blood can be characterized by both high levels of T cell-receptor (TCR) excision circle DNA (TREC)^13^ and the expression of the transmembrane marker, CD31.^11^ CD31 is an immunoglobulin-like molecule with an important role in modulation of T-cell responses,^14, 15^ and stimulation of the TCR leads to loss of CD31 expression.^16^ In adults and older children, a proportion of naïve CD4^+^ T cells do not express CD31, have relatively low levels of TREC, and are designated ‘central naïve' T cells. These factors indicate that they have undergone proliferation following exodus from the thymus.^12^ It has been suggested that, as the thymic output of naïve T cells reduces with age, the formation of central naïve T cells in the periphery have a role in the maintenance of an adequate naïve T-cell population.^11, 17^ Although the central naïve CD4^+^ T cells are present in significant proportions in adults and older children, little is known about the levels at birth or during the first postnatal year.

Treg cells that have a critical role in the control of self-tolerance and the development of acquired immunity^18^ can be differentiated according to a naïve phenotype and the transcription factor, forkhead box P3 (FoxP3).^19^ Miyara *et al.*^20^ demonstrated that the resting Treg (rTreg) and activated Treg (aTreg), two phenotypically and functionally distinct thymic-derived Treg subpopulations, could be discriminated on the basis of the degree of expression of FoxP3, and the presence or absence of CD45RA. Their study and subsequent work have shown age- and disease-related differences in the proportion of rTreg and aTreg,^21, 22^ which is consistent with the concept that they are a useful measure of Treg maturation and function. As the overall balance between Treg and T helper cells is linked to the development of allergic disease and other noncommunicable diseases,^23, 24^ measures of Treg proportions early in life may be particularly informative. Current studies of Treg at birth and during infancy are restricted to flow cytometric measures defined by expression of CD4^+^/CD25^+^/FoxP3^+^;^25, 26^ however, this combination of markers does not clearly differentiate the naïve rTreg from activation-induced FoxP3^+^ T cells.

The aim of this study, therefore, was to describe, in a population-derived cohort of infants, the natural history of key CD4^+^ T-cell subsets during the first postnatal year and to evaluate the cell percentages according to gender. Specifically, we sought to evaluate longitudinal age- and sex-related changes in naïve CD4^+^ T cells (thymic and central), and naïve Treg subgroups.

## Results

### Characterization of CD4^+^ T-cell subsets

**Figure 1:**
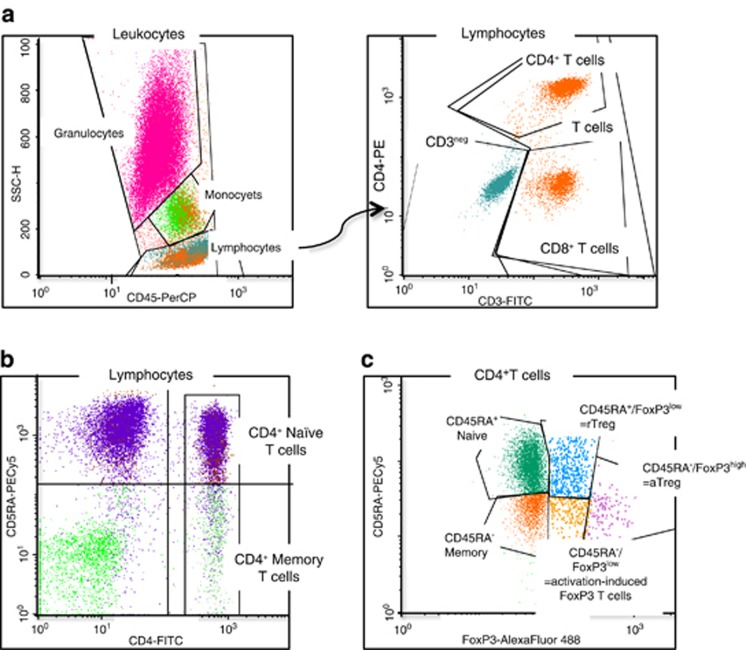
Gating for flow analysis of T-cell subsets. (**a**) CD3^+^ and CD4^+^ T cells. Whole blood cells were surface stained with fluorochrome-labeled monoclonal antibodies to CD3, CD4 and CD45. Lymphocytes were gated on the basis of CD45 and granularity (side scatter). CD3^+^ and CD4^+^ T cells were selected on the basis of CD3 and/or CD4 expression. (**b**) CD4^+^ naive T cells. MNCs were surface stained with fluorochrome-labeled monoclonal antibodies to CD4 and CD45RA. CD4^+^ T cells were gated, and naive CD4^+^ T cells were selected on the basis of CD45RA expression. (**c**) CD4^+^ Treg. MNCs were stained with fluorochrome-labeled monoclonal antibodies to CD4, CD45RA and FoxP3. Events were gated to the CD4^+^ T cells, and FoxP3^+^ subsets were selected on the basis of CD45RA and FoxP3 expression. CD45RA^+^FoxP3^low^, thymus-derived rTreg; CD45RA^−^FoxP3^high^, aTreg; CD45RA^−^FoxP3^low^, activation-induced FoxP3^+^ T cells.

All isolated mononuclear cells (MNCs) had a viability >99.5%. T-cell and CD4^+^ T-cell subsets were identified using specific antibodies and gating as shown in Figure 1.

#### Validation of thymic versus central naïve T-cell populations by TREC PCR analysis

**Figure 2:**
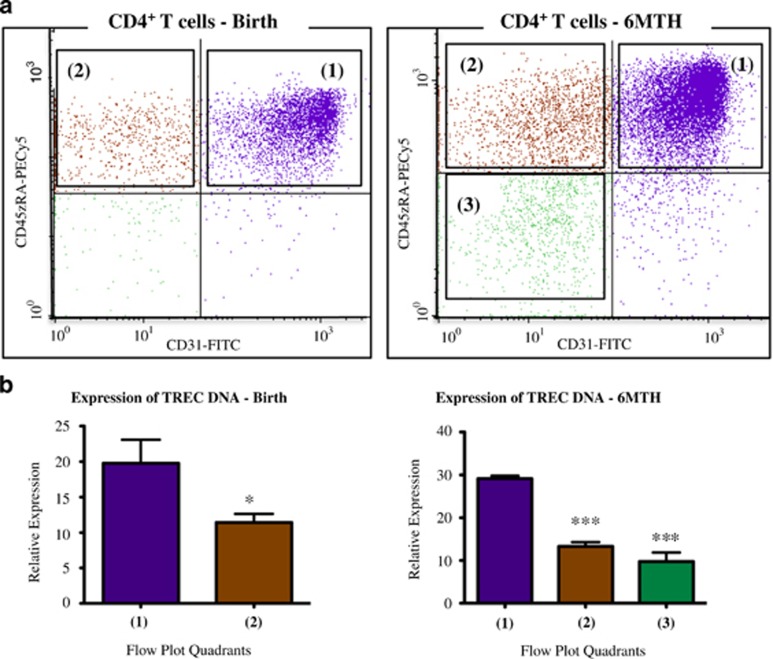
Validation of thymic and central naive CD4^+^ T-cell subsets. (**a**) MNCs were stained with fluorochrome-labeled monoclonal antibodies to CD4, CD31 and CD45RA. Cells were gated to the CD4^+^ population, and CD45RA^+^ naive cells were divided according to quadrant (1) (CD31^+^/CD45RA^+^) and quadrant (2) (CD31^−^/CD45RA^+^), with CD45RA^−^ cells in quadrant (3) (CD31^−^/CD45RA^−^). Representative flow plots at birth and 6 months are shown. (**b**) Relative expression of TREC DNA in sorted CD4^+^ T-cell populations from quadrants (1), (2) and (3). Thawed MNC samples from participants (*n*=5) at birth and 6 months were flow sorted and relative TREC expression was assessed. At birth, expression in quadrant (1) (CD31^+^/CD45RA^+^, thymic naïve) was greater than quadrant (2) (CD31^−^/CD45RA, central naïve; *P<0.05 paired *t*-test). Similarly, at 6 months, TREC expression in cells from quadrant (1) were greater than cells from either quadrant (2) or quadrant (3) (CD31^−^/CD45RA^−^, memory cells; ***P<0.0001, repeated measures analysis of variance). There was no difference in TREC expression of cells in quadrant (2) versus quadrant (3) at the 6-month time point.

Anti-CD31 was used to discriminate two subpopulations of naïve CD4^+^ T cells (Figure 2a). To confirm the thymic (CD31^+^/CD45RA^+^) and central (CD31^−^/CD45RA^+^) naïve phenotypes, target populations were flow sorted, genomic DNA extracted and relative TREC quantitated. TREC content was highest in the thymic naïve cells and significantly lower in the central naïve cells at both birth and 6 months (Figure 2b). Cells that had a non-naïve (memory) phenotype (CD31^−^/CD45RA^−^) were also sorted from the 6-month samples. The TREC DNA in these cells was similar to the central naïve cells (Figure 2b). TREC measures confirmed that the flow cytometric analysis correctly delineated thymic naïve and proliferating central naïve phenotypes.

### Longitudinal analysis of T-cell subsets over the first postnatal year

The distribution of each T-cell population was examined, and the mean percentages (with 95% confidence intervals (CIs)) at birth, 6 and 12 months are displayed in Table 1.

#### CD3^+^ and CD4^+^ T-cell subsets

**Figure 3:**
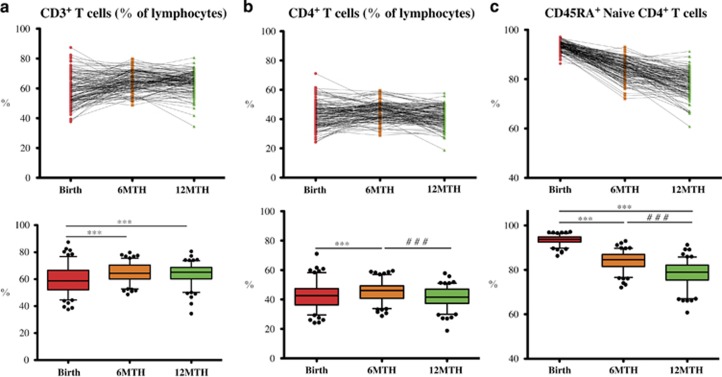
Longitudinal analysis of T-cell subsets. Longitudinal flow analysis of T-cell subsets at birth, 6 and 12 months (*n*=130 repeated measures) and represented by a line graph (top panel), and box and whisker plot (5–95 percentile, lower panel) for: (**a**) CD3^+^ T cells expressed as a percentage of the lymphocyte population. From birth to 6 months or 12 months, there was a mean increase (***P<0.0001), whereas from 6 to 12 months, there was no change (*P*=0.72). (**b**) CD4^+^ T cells expressed as a percentage of the lymphocyte population. From birth to 6 months, there was a mean increase (***P<0.0001), and then a mean decrease (^###^*P*<0.0001) from 6 to 12 months. (**c**) CD45RA^+^ naïve CD4^+^ T-cell subsets expressed as a percentage of the CD4^+^ T cells. There is a stepwise decrease over the first year, with a reduction from birth to 6 months (***P<0.0001), and a further decrease from 6 to 12 months (^###^*P*<0.0001).

Total T cells (CD3^+^) and CD4^+^ subsets (CD3^+^CD4^+^) were measured in whole blood as a percentage of the total lymphocyte population. There was a minor increase in the mean proportion of CD3^+^ T cells between birth and 6 months (5.4% (95% CI 3.6, 7.1)), and no further change from 6 to 12 months (Table 1). From birth to 6 months, there was a small increase in mean percentage of CD4^+^ T cells (2.5% (95% CI 1.1, 3.9)), and then a decrease from 6 to 12 months back to birth levels (3.7% (95% CI 2.4, 4.9)) (Table 1, Figures 3a and b).

#### Naive CD4^+^ T-cell populations

At birth, the CD4^+^ T cells were predominantly of a naïve (CD45RA^+^) phenotype. There was a stepwise decrease in mean CD45RA expression over the 12-month period (birth to 6 months, 9.4% (95% CI 8.7, 10.1), and 6–12 months, 5.7% (95% CI 4.8, 6.6)) with some individual variation noted (Table 1,Figure 3c).

#### Thymic and central naïve CD4^+^ T cells

**Figure 4:**
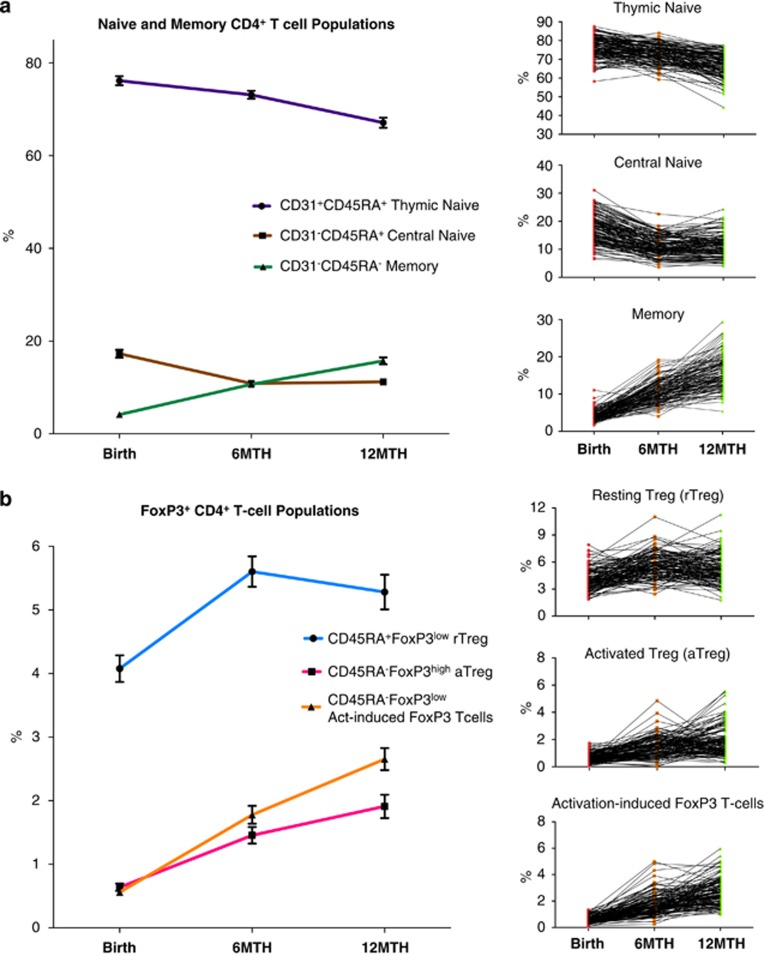
Longitudinal analysis of thymic and central naive CD4^+^ T cells and Treg subsets. (**a**) Longitudinal flow analysis of thymic, central naive and memory CD4^+^ populations as a percentage of CD4^+^ T cells (*n*=130 repeated measures, line graph with mean±95% CI). There was a minor drop (*P*<0.0001) in thymic naive CD4^+^ T cells between birth and 6 months, and a further decrease, from 6 to 12 months. There was a drop in central naive CD4^+^ T cells (*P*<0.0001) between birth and 6 months, but no change in percentage between 6 and 12 months (*P*=0.18). The CD4^+^ T-cell memory population increased (*P*<0.0001)) from birth to 6 months with a further increase to 12 months (*P*<0.0001). (**b**) Longitudinal analysis of FoxP3^+^ CD4^+^ T-cell subsets during the first year of life expressed as a percentage of CD4^+^ T cells (*n*=130 repeated measures, line graph with mean±95% CI). There was a marked increase (*P*<0.0001) in rTreg from birth to 6 months, and then a slight reduction between 6 and 12 months (*P*<0.032). There was a greater than twofold increase (*P*<0.0001) in both aTreg and activation-induced FoxP3^+^ CD4^+^ T cells from birth to 6 months, and a further increase in both populations (*P*<0.0001) between 6 and 12 months.

We next investigated changes in the percentages of the populations of thymic naïve, central naïve and memory CD4^+^ T cells. There was a small decrease in the mean proportion of thymic naïve CD4^+^ T cells between birth and 6 months (3.0% (95% CI 2.1, 4.0)), followed by a more marked decrease between 6 and 12 months (6.0% (95% CI 5.1, 7.0)). The percentage of central naïve CD4^+^ T cells (CD31^−^/CD45RA^+^) decreased from birth to 6 months (6.4% (95% CI 5.8, 7.0)), but there was no change in percentage between 6 and 12 months. In contrast, the mean percentage of CD4^+^ T cells with a memory phenotype (CD31^−^/CD45RA^−^) increased from birth to 6 months (6.5% (95% CI 6.0, 7.0)) and to 12 months (5.1% (95% CI 4.4, 5.8)) (Table 1,Figure 4a).

#### FoxP3^+^ populations within the CD4^+^ T cells

We characterized the CD4^+^ T cells according to the method of Miyara *et al.*,^10^ with the CD45RA^+^ FoxP3^low^ population defined as thymus-derived rTreg, the CD45RA^−^ FoxP3^high^ cells designated as activated Treg (aTreg) and CD45RA^−^ FoxP3^low^ as the activation-induced FoxP3^+^ CD4^+^ T cells (Figure 3a). There was a marked increase in mean rTreg from birth to 6 months (1.5% (95% CI 1.2, 1.8)), and then a small reduction in this population between 6 and 12 months (0.3% (95% CI 0.0, 0.6)). The aTreg, which are derived primarily from the rTreg, increased more than twofold from birth to 6 months, and then underwent a further increase between 6 and 12 months (0.45% (95% CI 0.2, 0.7)). The activation-induced FoxP3^+^ CD4^+^ T cells also increased in a linear fashion from birth to 6 months (1.25% (95% CI 1.1, 1.4)) and from 6 to 12 months (0.90% (95% CI 0.7, 1.1); Table 1, Figure 4b).

#### Investigation of CD4^+^ T-cell ontogeny over the first postnatal year by gender

We evaluated the longitudinal changes in the CD4^+^ T-cell population percentages according to gender. There was no difference in the proportion of CD3^+^, CD4^+^ T cells, naïve CD4^+^ T cells or FoxP3^+^ populations between males and females; however, for thymic naïve CD4^+^ T cells, males had a lower percentage at all time points compared with females (overall, −1.8% (95% CI −3.4, 0.3)) and a higher percentage of central naïve CD4^+^ T cells at all time points (overall, 2.6% (95% CI 1.4, 3.7); Table 2).

### Overall changes in composition of the CD4^+^ T-cell compartment

**Figure 5:**
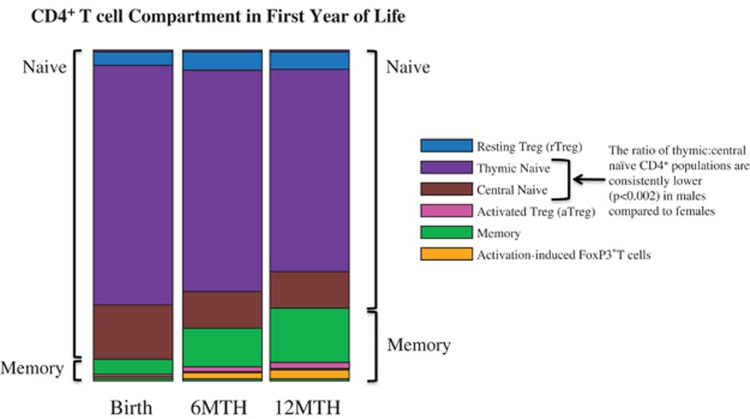
Diagrammatic overview of proportional changes in naive and Treg subsets within the CD4^+^ T-cell compartment during the first year of life.

We combined measures of the various CD4^+^ phenotypes assessed in this study into a composite view of the CD4^+^ T-cell compartment in which changes in subset proportions are simply visualized (Figure 5).

## Discussion

This is the first study to report multiple longitudinal CD4^+^ T-cell phenotypes from a large population of infants born at/near term, sampled from a population-derived cohort, at three time points during the first year of postnatal life. At birth, almost all CD4^+^ T cells are naïve, but a significant proportion of these have undergone proliferation (that is, have reduced TREC and are CD31^−^). In the first 6 months, the naïve population decreases and the non-naïve (memory) CD4^+^ T cells increase, in concert with a decrease of the subset of central naïve CD4^+^ T cells. There are gender differences in the birth thymic and central naïve CD4^+^ T-cell populations and these persisted throughout the first postnatal year. At birth, both rTreg and aTreg are present in the CD4^+^ T-cell compartment and their proportions increase during the first year of life.

Despite some individual variation in percentages, a small mean increase of both CD3^+^ and CD4^+^ T-cell subsets was observed in the first 6 months of life. Previous studies that lack longitudinal data have found similar T-cell population percentages at the three time points,^6, 7^ whereas de Vries *et al.*^5^ showed, in a longitudinal study of 11 infants, a comparable increase in the proportion of CD3^+^ but not CD4^+^ T cells. Among those infants, the frequency of CD45RA^+^ CD4^+^ T cells at birth was only 70% however, we clearly show in a large population sample that, consistent with other studies,^28, 29^ the CD4^+^ T cells at birth are primarily naïve (>93%) and that over the subsequent 12 months, there is a gradual reduction in the naïve phenotype, reflecting the development of the infants' adaptive immune response.^30^ A similar reduction in the naïve CD4^+^ T-cell population has previously been reported in cross-sectional studies;^7^ however, to further develop these data, we analyzed the naïve CD4^+^ T cells subsets: CD31^+^, thymic naïve; and CD31^−^, central naïve. The CD31^+^ population represented the majority of naïve cells at all ages investigated and had the highest relative expression of TREC; the signature marker of thymic naïve cells.^13, 31^ Interestingly, contrary to previous reports,^31, 32^ we found that at birth, the CD31^−^ central naïve population represented a fifth of the neonatal naïve CD4^+^ population, nearly double the level seen at 6 and 12 months, and similar to that observed in adults.^33^ Thus, it appears that a substantial proportion of central naïve CD4^+^ T cells is formed during gestation. The immunoglobulin-like molecule, CD31, is known to be shed following TCR engagement with major histocompatibility complex,^16^ but not following *in vitro* cytokine activation,^34, 35^ which suggests that further studies are required to determine which *in utero* signals stimulate the proliferation of naïve CD4^+^ T cells. It has been suggested that low-affinity self-peptide–major histocompatibility complex interactions may be involved in the generation of the central naïve cells, and that they have a role in the maintenance of the overall naïve population.^36^ The central naïve CD4^+^ T cells we observed at birth may at least in part explain the reported rapid proliferative responses of cord blood MNCs to various antigens seen in other studies.^32^

Sexual dimorphism was observed with male newborns having an increased proportion of CD31^−^ central naïve CD4^+^ T cells (and decreased thymic naïve) at birth, and this difference was maintained throughout the postnatal year. This indicates that a greater number of naïve CD4^+^ T cells in male infants are in a proliferative state and no longer express the immature thymic phenotype.^12^ An age-related reduction in TREC expressing thymic naïve T cells has previously been reported in the elderly, and the rate of this reduction appears to be greater in males.^37^ The gender differences reported here at birth are in keeping with this observation, and these might be the earliest signs of sexual dimorphism in immune function.^38^

The small but vital CD4^+^ subpopulation of Treg has important suppressive functions that limit autoimmune and allergic responses. We used specific cellular markers that provided longitudinal measures of the thymic-derived and naïve rTreg.^20^ Within the Treg population, rTreg predominated at birth and then increased during the first 6 months of life. This indicated accelerated Treg thymic activity in contrast to the overall fall in the entire population of naïve CD4^+^ T cells. After 6 months, the rTreg population started to decrease, a trend that presumably continues with increasing age. Indeed, by adulthood, the proportion of rTreg has dropped to 0.5–1.0%^20^ from an estimated 4.1% at birth observed in this study. The aTreg and activation-induced FoxP3^+^ CD4^+^ T cells increased throughout the first year of life. The population of aTreg is reported to be primarily derived from vigorously proliferating rTreg cells and it displays suppressive properties equal to that of rTreg,^10^ but are terminally differentiated and undergo apoptosis following suppressive activity.^20^ We demonstrated that by 12 months, aTreg comprise (on average) 24% of the total Treg population. Their memory phenotype suggests that they have undergone antigen recognition and may have a tailored immune-suppressive response.^39^ Indicative of their clinical importance, the frequency of aTreg has been reported to be modulated in aged donors,^20^ as well as disease states including active sarcoidosis,^20^ chronic obstructive pulmonary disease,^21^ type 1 diabetes^40^ and systemic sclerosis.^22^ Our analysis is the first to report rTreg populations in an infant cohort and may define new measures for immune outcomes related to infant disease.^41^

## Conclusion

The results from this prospective cohort^27^ demonstrate the normal ontogeny of CD4^+^ T-cell populations during the first 12 months of life, a period when immune responses may be susceptible to environmental influence. These findings provide insight into normal immune development and the ontogeny of naïve CD4^+^ during the first year of life.

## Methods

### Study design

The Barwon Infant Study is a population-derived birth cohort (*n*=1074) with antenatal recruitment in Victoria (Australia) that has been established to investigate the early life origins of a range of noncommunicable diseases in the modern environment.^27^ Infants born before 32 completed weeks gestational age were excluded, as were those with a serious illness, or major congenital malformation and/or genetically determined disease. Blood samples were collected from the participants at birth (cord blood), 6 and 12 months of age. Here we present data from the first 130 infants (68 females and 62 males) born >36 weeks gestation that had flow cytometric measures completed on freshly collected blood samples at all three time points.

### Blood sampling and processing

Umbilical cord blood was collected from the participants at the Geelong Hospital (public) and at the St John of God Hospital (private), Geelong, Victoria. Where possible, blood was collected before placenta delivery, by syringe. Up to 20 ml of cord blood was added to a 50-ml Falcon tube that already contained 20 ml of RPMI medium (Gibco, Life Technologies, Mulgrave, VIC, Australia) and 200 IU preservative-free sodium heparin (Pfizer, Australia Pty Ltd, West Ryde, NSW, Australia). At 6 and 12 months, venous peripheral blood was added to a 10-ml Falcon tube containing 100 IU preservative-free sodium heparin. All bloods were maintained at room temperature on a roller and processed within 18 h of collection. An aliquot (100 μl) of the whole blood was removed for measurement of the proportion of T cells and CD4^+^ T cells, and the remaining blood sample was processed to isolate plasma and MNCs. The MNCs were isolated from the whole blood sample using the density-gradient centrifugation (Lymphoprep, AxisShield, Oslo, Norway), and cell number and viability were assessed by Trypan Blue staining. Approximately 1–4 × 10^5^ MNCs were used for analysis of the CD4^+^ T-cell subsets.

### Characterization of CD4^+^ T-cell populations by flow cytometry

Antibodies were purchased from BD Biosciences (San Jose, CA, USA), and cells were stained to evaluate the relative frequency of T cells and CD4^+^ T-cell subpopulations. Isotype controls were used to set up the instrument and the positive gating, and these settings maintained throughout. To determine the percentage of CD3^+^ and CD4^+^ T cells, a sample of whole blood was stained with antibodies to CD3-FITC, CD4-PE and CD45-PerCP for 15 min before lysis of the red blood cells (BD FACS Lysing Solution, BD, North Ryde, NSW, Australia), phosphate-buffered saline wash and formalin fixation.

To measure specific subpopulations of CD4^+^ T cells, MNCs were stained either with (a) antibodies to CD4-FITC, CD31-PE and CD45RA-PECy5 (naïve CD4^+^ T cells and thymic and central naïve subsets); or (b) antibodies to CD4-PE and CD45RA-PECy5 (Treg subsets); washed in phosphate-buffered saline and formalin fixed. After overnight fixation, cells from (b) were permeabilized (0.5% Tween) and stained with anti-FoxP3 Alexa Fluor 488. All samples were analyzed by the 3-channel flow cytometry (FACSCalibur, Becton Dickinson).

### Sorting of naïve CD4^+^ cell populations

Cryopreserved MNCs from five participants (birth and 6 month samples from each participant) were sorted for TREC analysis. In brief, MNCs were thawed and washed in RPMI with 10% fetal calf serum, before being stained with antibodies to CD4-FITC, CD31-PE and CD45RA-PECy5 to discriminate three main populations using flow cytometric quadrant analysis. Cells were gated to the CD4^+^ population of lymphocytes and divided into flow plot quadrant (1), CD31^+^/CD45RA^+^; quadrant (2), CD31^−^/CD45RA^+^; and quadrant (3), CD31^−^/CD45RA^−^. The cells from quadrants (1) and (2; birth) and from quadrants (1), (2) and (3; 6 months) were sorted using a MoFlo (Beckman Coulter, Lane Cove, NSW, Australia) and the relative TREC expression was determined. A distinct population was not evident in remaining quadrant (CD31^+^/CD45RA^−^).

### Relative expression of TREC

Genomic DNA was extracted from each of the sorted CD4^+^ T-cell subsets (Qiagen AllPrep Mini Kit, Qiagen Sciences, Germantown, MD, USA) and measured using a NanoDrop 1000 (Thermo Scientific, Wilmington, DE, USA). All the DNA samples were diluted to a concentration of 5.0 ng μl^−1^, and a total of 15 ng used in a real time PCR reaction (Applied Biosystems 7500 Fast Real Time PCR System, Life Technologies) to amplify the following: (i) *TCR delta J3* section of excision circle DNA (forward primer: 5′-CTCAGGTCCTTAGAAAGCCT-3′ and reverse primer: 5′-CTCTTGGGTCACAAGTACAG-3′), and (ii) the single-copy *36B4* human sequence (forward primer: 5′-CAGCAAGTGGGAAGGTGTAATCC-3′ and reverse primer: 5′-CCCATTCTATCATCAACGGGTACAA-3′) using Sybr Green chemistry. The C_T_ value for *36B4* gene was subtracted from the C_T_ value for the *TCR delta J3* gene region (delta C_T_), and an arbitrary value for relative expression of TREC DNA in each group was calculated by measuring the power to base 2 of delta C_T_.

### Statistical analysis

Flow cytometric data from 130 Barwon Infant Study infants at the three time points were collected (birth, 6 and 12 months), with no missing values. Estimates of differences in the mean percentages of various T-cell populations over the first postnatal year were obtained using regression models with generalized estimating equations employed to account for repeated measures at the individual level. Results of the PCR comparative gene analysis of TREC DNA (performed in a small subgroup of infant samples) were analyzed using a paired *t*-test (birth) or repeated measures analysis of variance (6 months). All statistical analyses were conducted using Stata/IC 13.0 for Mac (StataCorp LP, College Station, TX, USA).

## Figures and Tables

**Table 1:** Mean percentages (with 95% CIs) of T-cell subtypes during the first year of life (*n*=130 infants)

*Cell populations*	*First year of life*					
	*Birth*		*6 months*		*12 months*	
	*Mean (%)*	*95% CI*	*Mean (%)*	*95% CI*	*Mean (%)*	*95% CI*
* As % of lymphocytes*						
* *CD3^+^ T cells	59.3	57.6, 61.1	64.7	63.5, 65.8	64.1	62.9, 65.3
* *CD4^+^ T cells	42.7	41.3, 44.2	45.2	44.1, 46.3	41.6	40.4, 42.7
						
* As % of CD4*^*+*^ *T cells*						
* *CD45RA^+^ naïve	93.5	93.1, 93.8	84.1	83.3, 84.8	78.3	77.4, 79.3
* *CD31^+^ thymic naïve	76.1	75.2, 77.1	73.1	72.3, 74.0	67.1	66.0, 68.2
* *CD31^−^ central naïve	17.4	16.5, 18.2	10.9	10.3, 11.5	11.2	10.5, 11.9
* *CD45RA^−^/CD31^−^ memory	4.1	3.9, 4.4	10.7	10.2, 11.2	15.7	15.0, 16.5
* *rTreg, CD45RA^+^FoxP3^low^	4.1	3.9, 4.3	5.6	5.4, 5.8	5.3	5.0, 5.6
* *aTreg, CD45RA^−^FoxP3^high^	0.72	0.66, 0.79	1.46	1.32, 1.59	1.91	1.72, 2.10
* *Activation-induced FoxP3 T cells (CD45RA^−^FoxP3^low^)	0.53	0.53, 0.63	1.78	1.63, 1.92	2.65	2.48, 2.83

Abbreviations: aTreg, activated T regulatory cell; CI, confidence interval; rTreg, resting T regulatory cell.

**Table 2:** Mean percentages of thymic and central naive T-cell subtypes during the first year of life according to gender (*n*=62 males, *n*=68 females)

	*Birth*		*6 months*		*12 months*		*Overall (longitudinal)^a^*	
	*Male*	*Female*	*Male*	*Female*	*Male*	*Female*	*Male*	*Female*
*As % of CD4*^*+*^ *T cells*								
CD31^+^ thymic naive	74.9	77.3	72.1	74.1	66.6	67.6	−1.8 (95% CI, −3.4, 0.3)	
* P-*value	0.018		0.024		0.37		0.021	
CD31^−^ central naive	18.5	16.3	12.4	9.5	12.5	10.1	2.6 (95% CI, 1.4, 3.7)	
* P*-value	0.011		<0.001		<0.001		<0.001	
Thymic/central naive ratio	4.6:1	5.2:1	6.5:1	8.7:1	6.0:1	7.7:1	−1.2 (95% CI, −1.9, 0.5)	
* P-*value	0.056		<0.001		0.001		0.002	

Abbreviation: CI, confidence interval.

a Mean difference (male to female).
